# 1151. Clinical Characteristics of Persistent Staph Aureus Bacteremia in Children

**DOI:** 10.1093/ofid/ofab466.1344

**Published:** 2021-12-04

**Authors:** Nicholas Venturelli, Palak Bhagat, Allison Nelson, Madan Kumar

**Affiliations:** 1 University of Chicago Medical Center, Chicago, Illinois; 2 University of Chicago Medicine, Chicago, IL; 3 University of Chicago Medicine Comer Children’s Hospital, Geneva, IL; 4 University of Chicago, Chicago, IL

## Abstract

**Background:**

Persistent *Staphylococcus aureus* bacteremia (pSAB) is a poorly defined entity, but associated with significant morbidity and mortality in children. We aim to better describe the epidemiological features of this clinical entity.

**Methods:**

We performed a retrospective case series analysis of pediatric patients with pSAB at a single center children’s hospital using electronic medical data from 2016 – 2020. Bacterial persistence was defined as culture growth > 72 hours after first blood culture.

**Results:**

Twenty-two patients with pSAB were included in the analysis. Sources of persistent infection were endovascular infection (n=11, 50%), osteoarticular infection (n=6, 27%,), isolated central line associated blood stream (n=4, 18%), isolated skin and soft tissue infection (n=2, 9%), and no known primary infectious site (n=1). Methicillin resistance occurred in 41% (n=9) of cases of pSAB. Total duration of therapy varied, with a median of 4 weeks from negative cultures (range of 2 – 8 weeks). Total days of positive cultures in pSAB were not significantly associated with methicillin susceptibility of the bacterial isolate, use of double gram-positive coverage, nor presence of a central venous catheter. Use of double gram-positive coverage occurred in 50% of cases with a mean duration of therapy of 11 days, most frequently in cases of septic thrombophlebitis (Table 1). Rifampin and gentamicin were the most commonly used agents.

Table 1. Clinical Characteristics of Children Treated with Double Gram-Positive Coverage

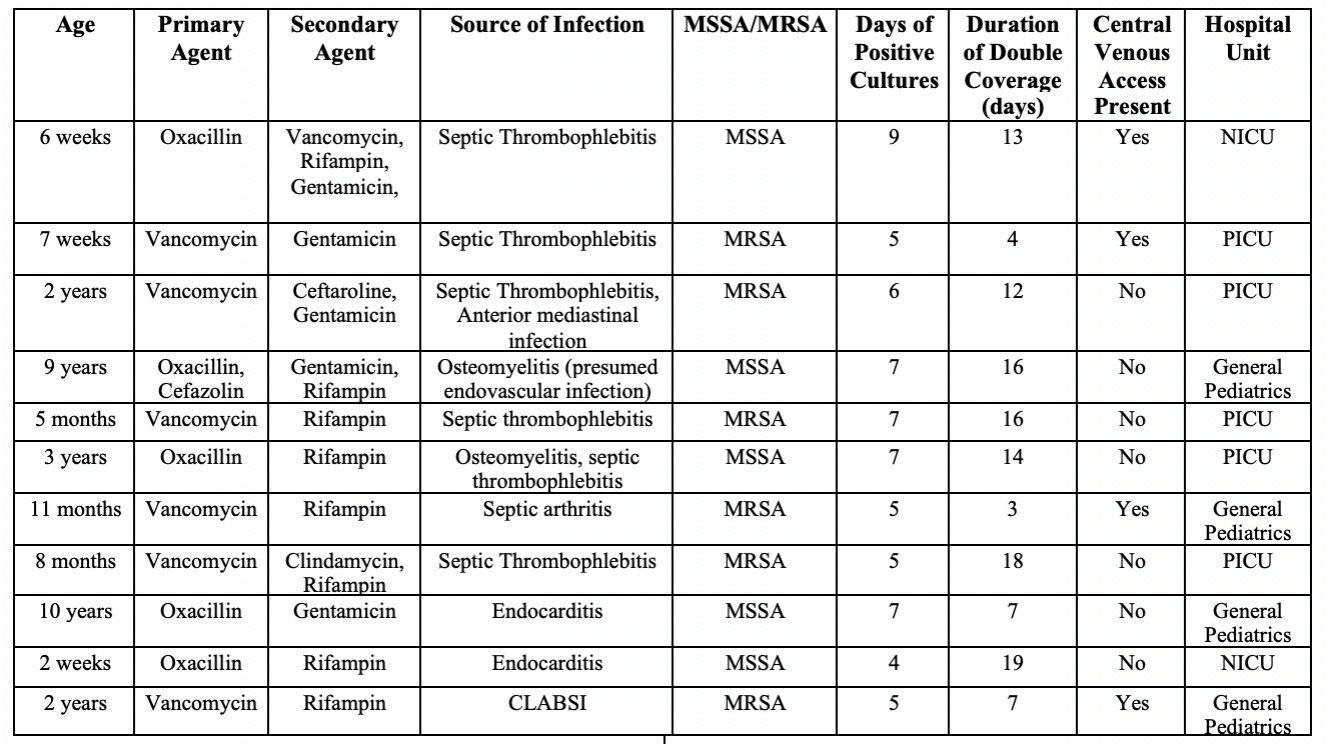

**Conclusion:**

Children presenting with persistent S. aureus bacteremia present with a heterogenous group of underlying conditions and epidemiological features. While pediatric recommendations for double gram-positive coverage for synergy have not been established, their use for pSAB is common, especially in endovascular infections where culture persistence is often an expected outcome. Further research should examine risk factors for pSAB and define optimal treatment modalities and duration.

**Disclosures:**

**All Authors**: No reported disclosures

